# A multi-responsive healable supercapacitor

**DOI:** 10.1038/s41467-021-24568-w

**Published:** 2021-07-14

**Authors:** Haili Qin, Ping Liu, Chuanrui Chen, Huai-Ping Cong, Shu-Hong Yu

**Affiliations:** 1 Anhui Province Key Laboratory of Advanced Catalytic Materials and Reaction Engineering, School of Chemistry and Chemical Engineering, Hefei University of Technology, Hefei, P. R. China; 2 Division of Nanomaterials and Chemistry, Hefei National Laboratory for Physical Sciences at Microscale, Institute of Energy, Hefei Comprehensive National Science Center, Department of Chemistry, Institute of Biomimetic Materials & Chemistry, University of Science and Technology of China, Hefei, P. R. China

**Keywords:** Supercapacitors, Materials for energy and catalysis, Polymers

## Abstract

Self-healability is essential for supercapacitors to improve their reliability and lifespan when powering the electronics. However, the lack of a universal healing mechanism leads to low capacitive performance and unsatisfactory intelligence. Here, we demonstrate a multi-responsive healable supercapacitor with integrated configuration assembled from magnetic Fe_3_O_4_@Au/polyacrylamide (MFP) hydrogel-based electrodes and electrolyte and Ag nanowire films as current collectors. Beside a high mechanical strength, MFP hydrogel exhibits fast optical and magnetic healing properties arising from distinct photothermal and magneto-thermal triggered interfacial reconstructions. By growing electroactive polypyrrole nanoparticles into MFP framework as electrodes, the assembled supercapacitor exhibits triply-responsive healing performance under optical, electrical and magnetic stimuli. Notably, the device delivers a highest areal capacitance of 1264 mF cm^−2^ among the reported healable supercapacitors and restores ~ 90% of initial capacitances over ten healing cycles. These prominent performance advantages along with the facile device-assembly method make this emerging supercapacitor highly potential in the next-generation electronics.

## Abstract (web summary)

Self-healing property is important for supercapacitors when powering the electronics, but designing devices that possess a universal healing mechanism remains challenging. Here, the authors achieve an optically, electrically, and magnetically-responsive self-healing device with integrated configuration.

## Introduction

Self-healing ability, widely found in biological tissues, is an attractive feature to repair internal or external damages automatically and allow structural and functional restorations, which is tightly related to long lifetime, good sustainability and high utilizing safety of artificial materials^1–3^. With the rapid development of wearable energy-storage devices, smart supercapacitors with self-healability have attracted particular research interests as they can restore their capacitive performance in the case of mechanical and structural damages under bending or other deformations. Most of the reported healable supercapacitors have been fabricated by either employing an extra self-healing polymer layer to wrap/support the electrode^4–7^, or using an additional electrode patch combined with the self-recovered electrolyte^8^. These non-intrinsically self-healing configurations between two electrodes sandwiched with an indispensable electrolyte layer lead to unsatisfactory healing efficiency and low energy density. Therefore, it is of great practical significance to realize and maintain integrated current collector-electrode–electrolyte–electrode-current collector configuration during the device fabrication and after mechanical fractures^9^. Facing with these challenges, the creation of effective healing motifs for design and construction of an omni-healable supercapacitor is highly desirable and important.

Until now, various healing mechanisms have been exploited to fabricate self-healing materials, including incorporation/release of curing agents^1^, and introduction of dynamic/reversible covalent or noncovalent interactions as the mobile phases^2,10–13^. Among them, dynamic metal-ligand coordination interactions have demonstrated their effectiveness in constructing smart materials with the repeated self-healing capability and enhanced mechanical property simultaneously because of their modest binding strength and adjustable stimuli-responsive thermodynamic equilibrium^14–16^. Recently, we have reported the synthesis of robust and smart nanocomposite hydrogels with great self-healing capability based on reversible gold-thiolate (Au–SR) coordination chemistry under the stimulus of near-infrared (NIR) laser^17^. Compared with thermally healable systems, the stimulus of light is undamaged to materials through exclusively exposing and healing the fractured region^14,18^. Moreover, photothermal property of noble metal can dissociate the metal-ligand motifs effectively, achieve surface reconstruction of coordination bonds, and therefore heal the fractures.

Meanwhile, magneto-thermal behavior, a fascinating origin of heat under a magnetic field, has been intensely studied in magnetic hydrogels composed of magnetic nano- or microparticles within the polymer hydrogel matrix^19–22^. So far, significant progress has been mainly made in the bio-fields of remotely controllable drug release and hyperthermal therapy^21,23,24^. In contrast, the magneto-thermal induced healing motifs have not been reported, although it is well accepted as a remotely controllable, homogeneous, and undamaged stimulus. One challenge is involved in achieving uniform distribution of magnetic nanoparticles in the hydrogel and preventing them from diffusing out of the matrices. Intelligently developing magneto-thermal triggered healing mechanism rather than the simple magnetic motion also remains great challenging^5,25,26^.

Herein, a kind of magnetic Fe_3_O_4_@Au/polyacrylamide (PAM) (MFP) hydrogel has been fabricated by chemically crosslinking disulfide bond-functionalized Fe_3_O_4_@Au nanocomposites into the polymeric network. Owing to effective energy dissipating mechanism from the homogeneously interconnected network structure, the MFP hydrogel demonstrates tough mechanical performance with large stretchability of 2250% its initial length and strong notch-insensitivity. Additionally, notable photothermal and magneto-thermal properties of the hydrogel enable the fabricated hydrogel with fast optical and remotely controllable magnetic healability relied on high-density dynamic Au–SR coordination bonds. Incorporating polypyrrole (PPy) nanoparticles into the MFP network as electrode, a supercapacitor prototype is assembled by sandwiching two MFP-PPy electrodes with a MFP hydrogel electrolyte and spray-coating silver nanowire (AgNW) films as current collectors. The synergistic effects of Au, Ag-SR bonds allow the assembled supercapacitor with integrated configuration to exhibit the largest areal capacitance up to 1264 mF cm^−2^ and a record-breaking device-level stretchability of 1200%, confirming it as one of the best performers among the flexible and healable supercapacitors. Impressively, this supercapacitor possesses intrinsically multi-responsive healing capability with ~90% of capacitance restored over 10 optical, electrical, and magnetic healing cycles, respectively. These great performances and simple device-assembly method promise the presented supercapacitors highly competitive in the next-generation wearable and portable electronic devices.

## Results

### Design and preparation of magnetic hydrogel

**Fig. 1:**
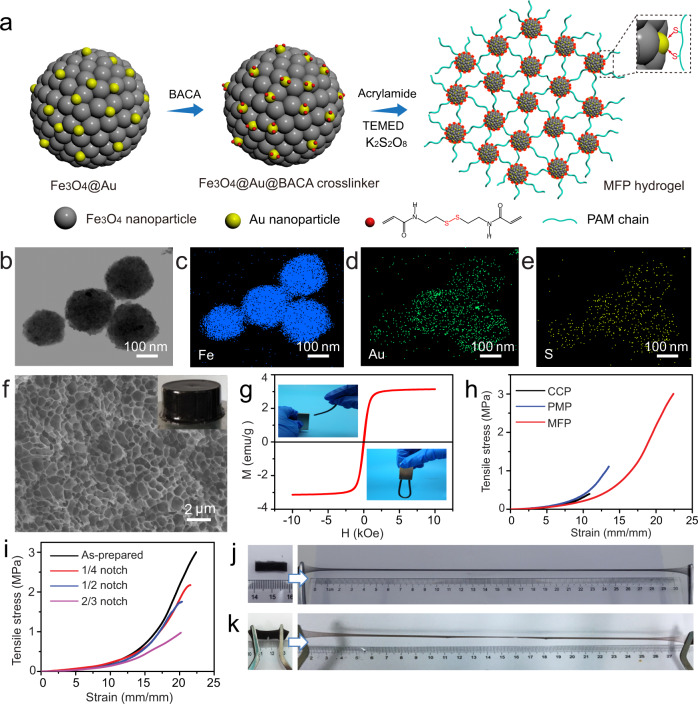
Preparation and characterization of MFP hydrogel. **a** Schematic illustrations of the preparation of MFP hydrogel. **b** TEM image of Fe_3_O_4_@Au@BACA composites. **c**–**e** Corresponding element mappings of Fe, Au, and S, suggesting the ultrafine coating of BACA. **f** SEM image of MFP hydrogel. The inserted photograph shows a columnar hydrogel. **g** Magnetic hysteresis loops of MFP hydrogel. The inserted optical images show magnetic response of MFP hydrogel with a magnet. **h** Tensile stress–strain curves of CCP, PMP, and MFP hydrogels. **i** Tensile stress–strain curves of MFP hydrogel notched with different sizes. Optical images show high stretchability of (**j**) MFP hydrogel and (**k**) the notched MFP hydrogel.

Figure 1a shows schematic illustrations of the preparation of MFP hydrogel. Typically, Fe_3_O_4_@Au composites were prepared through in situ reduction of Au nanoparticles on hydrothermally-synthesized Fe_3_O_4_ nanospheres^27^ at room temperature. The pre-obtained Fe_3_O_4_ nanospheres with a uniform diameter of ~200 nm were monodispersed and consisted of small nanoparticles with a size of 15 nm, behaving fast magnetic response when closed to a magnet (Supplementary Fig. 1a–d). Magnetic characterization indicated the superparamagnetism of Fe_3_O_4_ nanospheres with magnetization saturation value of 72.1 emu g^−1^ (Supplementary Fig. 2), confirming its nanoparticle-composed structure. With mild chemical reduction, the Fe_3_O_4_ nanospheres were decorated with large amount of Au nanoparticles with uniform size of ~5 nm, maintaining a monodispersed state (Supplementary Fig. 1e–h). X-ray diffraction (XRD) pattern and survey X-ray photoelectron spectroscopy (XPS) spectrum as well as core-leveled Au 4 *f* XPS spectrum of Fe_3_O_4_@Au composites proved the anchoring of Au nanoparticles onto Fe_3_O_4_ nanospheres (Supplementary Figs. 3 and 4). Notably, the obtained Fe_3_O_4_@Au composites held superparamagnetic characteristics with similar magnetization saturation value to Fe_3_O_4_ nanospheres, as shown from no remanence detected in the corresponding magnetic hysteresis loop (Supplementary Fig. 2). In order to get a kind of crosslinker for the hydrogel polymerization, the disulfide bond-ended N,N′-bis(acryloyl)cystamine (BACA) molecules were used and linked with Fe_3_O_4_@Au nanospheres via the Au–SR bonds, producing the Fe_3_O_4_@Au@BACA composites. As shown in transmission electron microscopy (TEM) image (Fig. 1b) and corresponding elemental mappings (Fig. 1c–e), the elements of Fe, Au, and S were homogeneously distributed in the Fe_3_O_4_@Au@BACA composites, demonstrating the success in binding BACA onto Fe_3_O_4_@Au. Subsequently, by serving Fe_3_O_4_@Au@BACA composites as the crosslinkers, the MFP hydrogel was fabricated through the free-radical polymerization of acrylamide monomer initiated by potassium persulfate (KPS) and accelerated by N,N,N′,N′-tetramethylethylenediamine (TEMED). Scanning electron microscopy (SEM) image showed that the MFP hydrogel delivered homogeneous network structure with the pore size of ~1 μm (Fig. 1f). The MFP hydrogel presented distinct superparamagnetism in the magnetic hysteresis loop and performed remarkable movement to the applied magnetic field (Fig. 1g), inheriting from the homogeneously dispersed Fe_3_O_4_@Au composites. For comparison, the conventional chemically crosslinked polymeric hydrogel was prepared by using BACA as chemical crosslinker without addition of Fe_3_O_4_@Au composites, denoted as CCP hydrogel. Because of the nature of irregular polymerization, the broadly distributed network structure was presented in the CCP hydrogel (Supplementary Fig. 5a). The other control sample of the physically incorporated magnetic PAM hydrogel, denoted as PMP hydrogel, was fabricated by using N,N′-methylene-bis-acrylamide (MBAA) rather than BACA via a similar method to the MFP hydrogel. Herein, the Fe_3_O_4_@Au nanocomposites were randomly aggregated in the PMP hydrogel network arising from no chemical bonds between MBAA and Fe_3_O_4_@Au composites (Supplementary Fig. 5b).

Resulting from chemically crosslinked Fe_3_O_4_@Au composites in the network, the MFP hydrogel exhibited notably mechanical performance. The tensile test quantified that the MFP hydrogel delivered a high tensile stress up to 3.1 MPa at an elongation of 2250% (Fig. 1h). The MFP hydrogel piece with the length of 2 cm could be stretched to a length of >30 cm without fracture (Fig. 1j). In sharp contrast, the control samples showed much weakened mechanical properties with the tensile stress of 0.5 MPa at the strain of 1100% for CCP hydrogel and 1.1 MPa at 1300% for PMP hydrogel (Fig. 1h), indicating the regularly crosslinked structural advantage in MFP hydrogel. Statistics showed much enhanced toughness of 17 MJ m^−3^ for MFP hydrogel, compared with that of 1.2 MJ m^−3^ for CCP and 3.5 MJ m^−3^ for PMP hydrogel (Supplementary Fig. 6). Impressively, the MFP hydrogel demonstrated remarkable notch-insensitivity property, as indicated from high stretchability of the notched gel piece in the optical image (Fig. 1k). As recorded in Fig. 1i, the MFP hydrogels notched with different sizes presented greatly ascended tensile stress when they were stretched until rupture. Even with a large notch of 2/3 of its original width, a large strain of 2050% was still delivered, corresponding to 91% of elongation of the original sample. Further comprehensive tests discovered that the MFP hydrogel system with varied content of Fe_3_O_4_@Au composites all behaved tough mechanical performance (Supplementary Fig. 7).

With increasing the content of Fe_3_O_4_@Au nanocomposites in the hydrogel network, the mechanical behavior was improved correspondingly. The content of composites was optimized to 2.0 mg mL^−1^, due to that the excessive nano-crosslinkers were not crosslinked in the polymeric network and weakened the hydrogel strength. Additionally, the MFP hydrogel showed strong compression-resistant capability. As seen from optical images (Supplementary Fig. 8), the highly compressed hydrogel column was rapidly recovered its initial state since the compressive force was released. However, it was observed that the columnar CCP hydrogel was broken into pieces and the PMP hydrogel was fractured when they were under the compression.

### Stimuli-triggered healing property of MFP hydrogel

**Fig. 2:**
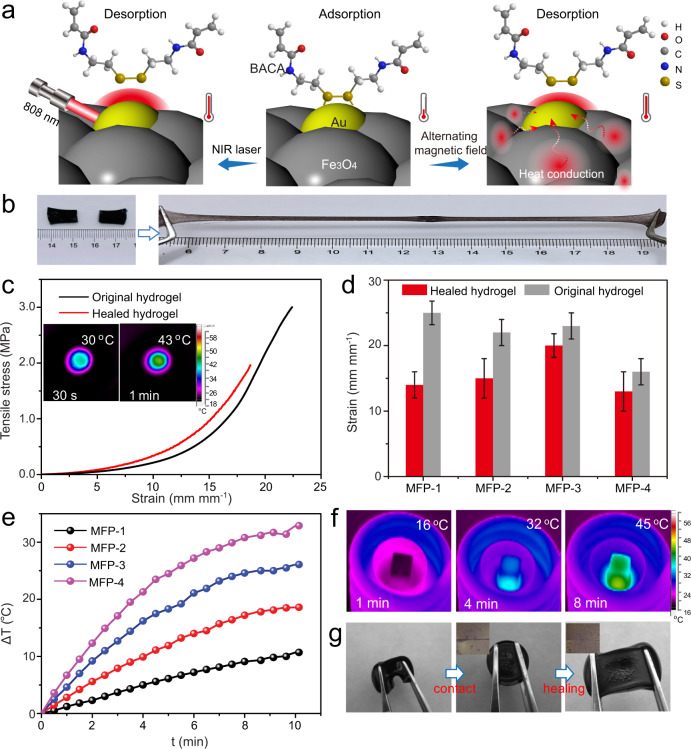
Stimuli-triggered healing property of MFP hydrogel. **a** Schematic illustrations of photothermal and magneto-thermal induced self-healing mechanisms. **b**–**d** NIR laser-induced self-healing property: **b** Optical images show high stretchability of the healed hydrogel piece. **c** Tensile stress–strain curves of original and healed hydrogels. The inserted images show time-dependent temperature changes of hydrogel. **d** Strains of original and healed hydrogels with different contents of Fe_3_O_4_@Au composites. Error bars show the SD with sample size of 3. **e**–**g** Magnetically induced self-healing property: **e** Time-dependent temperature changes of the cylindrical hydrogels with different contents of Fe_3_O_4_@Au composites under alternating magnetic field. **f** Infrared thermal images of the cylindrical hydrogel. **g** Optical images show stretchability of the healed hydrogel. The inserted optical images show the glued structure of the fractured interface.

Given that a large number of dynamic Au–SR coordination bonds were homogeneously incorporated in the network, the MFP hydrogel possessed strong healing ability through the reversible deformation/reformation of Au–SR bonds under the external stimulus^17^. It was demonstrated that high temperature could trigger the surface reconstruction on the basis of dynamic bonds on/off switching from the surface^28^. However, because of weak thermal conduction of the hydrogel and additional water loss-induced decrease of polymer mobility, little healing was observed when the damaged hydrogel pieces were placed in the oven even for a long time (Supplementary Fig. 9). As illustrated in Fig. 2a, owing to distinct photothermal property of Au nanoparticles and excellent magneto-thermal property of Fe_3_O_4_ nanospheres from the uniformly dispersed Fe_3_O_4_@Au composites, the MFP hydrogel was expected to be healed inspired by NIR laser or magnetic field. When two individual gel pieces were close in contact and the fractured region was exposed to the NIR laser, it was observed that they were healed in 2 min and could be stretched to a large strain without any crack (Fig. 2b). Temperature tests by an infrared thermal imager in the inserted images in Fig. 2c showed that the temperature was up to 43 °C under the NIR irradiation within 1 min. Reasonably, by improving the content of Fe_3_O_4_@Au composites in the hydrogels, more obvious increase in temperature was detected (Supplementary Fig. 10). Tensile stress–strain curves in Fig. 2c quantified a stretch of 1900% was maintained for the healed hydrogel, achieving a high healing efficiency of 86.3% calculated from the strain ratios between the healed and original samples. Furthermore, it was found that the healing efficiency of MFP hydrogel was dependent on the content of the incorporated Fe_3_O_4_@Au composites. As counted in Fig. 2d, when increasing the composite content from 0.4 to 4.0 mg mL^−1^, the extensibility of the healed hydrogel was 62.5%, 69.6%, 86.3%, and 81.8% of the initial length, respectively, owing to the increased density of Au–SR sites in the hydrogels. This rapid and high-efficient healing ability outperformed the previously reported self-healing hydrogels with 52–99% of healing efficiency in 24 h^29–31^. Furthermore, a MFP hydrogel piece and a CCP hydrogel piece were able to be healed together under NIR laser and behaved strong resistant to stretching deformation (Supplementary Fig. 11), fully confirming the dynamic nature and effectiveness of Au–SR bonds as healing motifs.

In addition to fast and efficient optical healability, the MFP hydrogel demonstrated remotely controllable magnetic healing performance arising from the surface reconstruction triggered by the heat conduction from Fe_3_O_4_ to Au nanoparticles. Notably, the nanoparticle-composed structure of the Fe_3_O_4_ nanospheres led to the enhanced magneto-thermal effect and activated the kinetics of Au–SR bonds. It was detected that the temperature change of the prepared Fe_3_O_4_ nanospheres was increased by 53 °C within 5 min when placed into the helical coil of the alternating magnetic field generator (Supplementary Fig. 12). When the Fe_3_O_4_@Au composites were crosslinked into the gel network, the MFP hydrogel remained excellent magneto-thermal performance. As shown in Fig. 2e, the temperature of the hydrogels delivered a time-dependent increasing characteristic and with improving the content of the magnetic composites, the real-time temperature increasing was more obvious. Even though 0.3 wt% of Fe_3_O_4_@Au was contained in the hydrogel, the temperature of the hydrogel column with a diameter of 5 cm was increased to 45 °C in 8 min as revealed by infrared thermal imaging system (Fig. 2f). As exhibited in Fig. 2g, when placing a columnar hydrogel with a large incision in the alternating magnetic field, the hydrogel was healed and could resist the external stretching force. The optical images in Fig. 2g clearly showed the fused structure of the damaged interface triggered by magnetic stimulus. To the best of our knowledge, this is the first demonstration of intrinsically magneto-thermal induced healing performance rather than magnetically driven physical contact.

### Assembly of omni-healable supercapacitor device

**Fig. 3:**
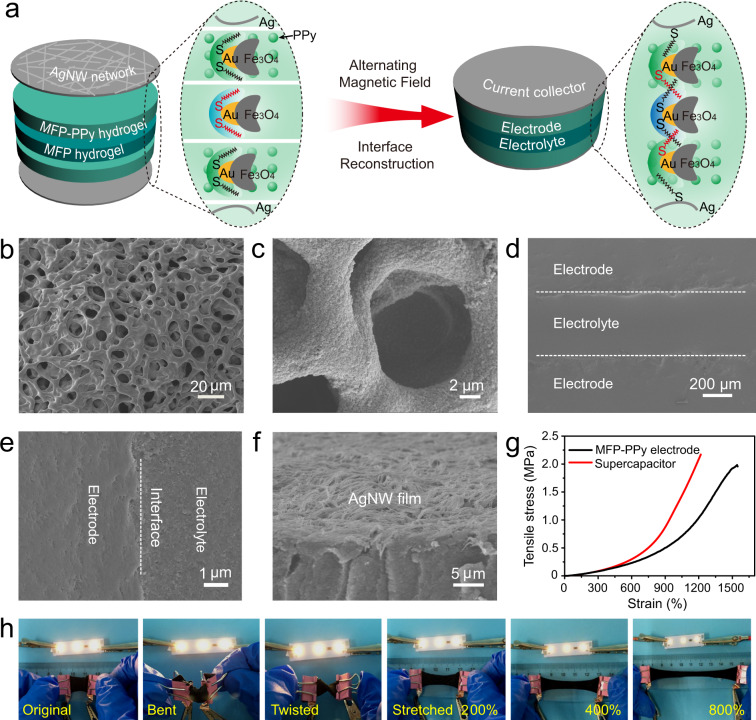
Assembly of the healable supercapacitor device. **a** Schematic illustrations of the magneto-thermal induced assembly of the supercapacitor by sandwiching two MFP-PPy hydrogel electrodes with a MFP hydrogel electrolyte and spraying AgNW film layers as current collectors. The device presents an integrated configuration owing to the metal-thiolate bond-triggered interface reconstruction under alternating magnetic field. SEM image (**b**) and enlarged SEM image (**c**) of MFP-PPy hydrogel electrode. **d** Cross-sectional SEM image of the supercapacitor. **e** Enlarged SEM image of the electrode/electrolyte interface of the supercapacitor. **f** SEM image of the AgNW film coated on the electrode. **g** Tensile stress–strain curves of the MFP-PPy hydrogel electrode and the assembled supercapacitor. **h** Optical images show good electrical conductivity of the supercapacitor at multiple deformations including bending, twisting, and stretching with high strains.

The high mechanical performance and excellent healability promised the MFP-based hydrogel as potential candidates in the assembly of flexible and healable supercapacitor. Especially, when employed for electrodes, the unique 3D architectures composed of molecular meshes around by polymer chains, interconnected channels and microporous structures offered great advantages on the infiltration of electrolyte and ion transfer in the electrochemical process, in contrast to the conventional electrode configurations (Supplementary Fig. 13a)^32^. To make an active electrode, the conductive polymer, PPy was in situ grown into the MFP framework through soaking the partly-dehydrated MFP hydrogel into the pyrrole solution and the subsequent polymerization initiated by Fe^3+^. SEM images showed that the MFP-PPy hydrogel maintained the interconnected network structure and a layer of PPy nanoparticles with the uniform size of 100 nm was coated on the wall of the compartment (Fig. 3b, c). Notably, after the PPy incorporation, the elegant porous structure was still presented in the composites. Compared with the original MFP hydrogel, the pore size of the MFP-PPy hydrogel was increased to 10 μm arising from the swollen state during the polymerization process. The MFP-PPy hydrogel exhibited high mechanical performance by delivering an elongation of 1650% (Fig. 3g). These structural advantages guaranteed the MFP-PPy hydrogel electrode fast transports of electron and electrolyte. As illustrated in Fig. 3a, to assemble a healable supercapacitor device, two pieces of MFP-PPy hydrogel electrodes were sandwiched by a MFP hydrogel electrolyte instead of the common PVA gel and AgNW films with the length of 60–100 μm and thickness of 1.5–2 μm were spray-coated on the electrodes to serve as current collectors (Supplementary Fig. 14a, b). Under the alternating magnetic field, the current collectors, electrodes, and electrolyte could be chemically soldered together by dynamic Ag, Au–SR crosslinkings owing to the magneto-thermal effect. Such an effective interface reconstruction resulted in an integrated device configuration as confirmed by the SEM images (Fig. 3d–f). Furthermore, the structural stability of the current collector embedded in the polymer network of the electrode had been investigated under a continuous stretching from 200% to 1000% strain. As shown from SEM images (Supplementary Fig. 14c–f), with increasing the strains up to a high value of 1000%, the AgNW network was straightened and no visible cracks were observed, indicating the excellent stretchability of the AgNW films as current collectors. More impressively, the mechanical test in Fig. 3g quantified that the assembled supercapacitor delivered a high break elongation of 1200%, indicating its superior stretchability among the reported flexible/stretchable supercapacitors^7,8,33–35^. The optical images in Fig. 3h revealed the good electrical conductivity of the assembled supercapacitor device under multiple deformations including bending, twisting, and stretching with large strains, demonstrating the stable network structure in the MFP-based hydrogels and the integrated device configuration. Based on above results, this work made a good demonstration on the magneto-thermal induced assembling of an intrinsically omni-healable supercapacitor based on synergistically controlling constituent, structure, and interfacial interaction of the electrodes, electrolyte, and current collectors.

### Multi-responsive healing performance of the supercapacitor

**Fig. 4:**
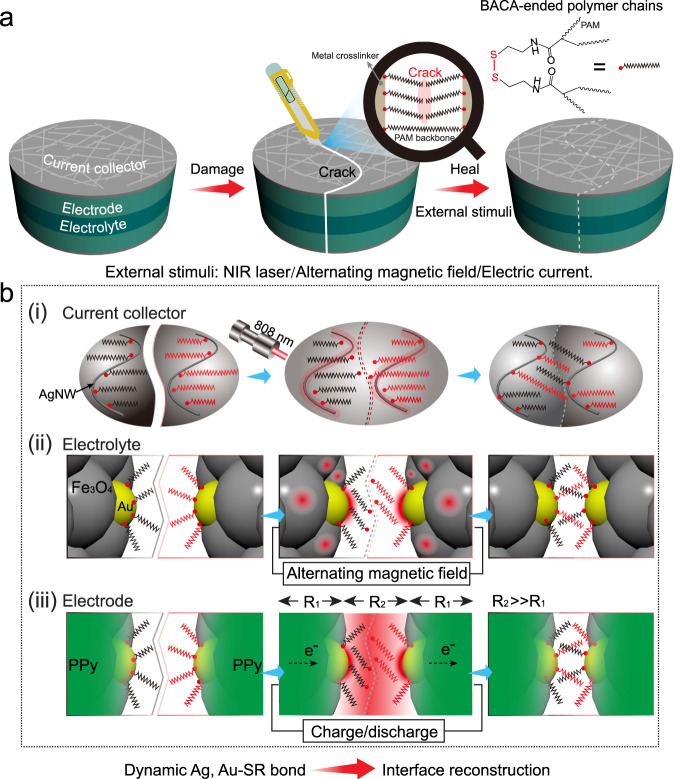
Schematic illustrations of self-healing mechanism of supercapacitor. **a** Scheme of the self-healing process. **b** Schematic illustrations for the healing mechanism of the damaged supercapacitor under the external stimuli, taking optical healing of current collector, magnetic healing of electrolyte, and electrical healing of electrode as examples. The dynamic Ag, Au–SR bond-induced interface reconstruction results in the healing of the crack region under the photothermal, magneto-thermal, and galvano-thermal effects.

Considering the intrinsically healable hydrogels employed as electrodes and electrolyte, the assembled supercapacitor showed great self-healing capability when got damaged (Fig. 4a). Typically, combined with the thermal-triggered reversible and dynamic feature of metal-thiolate interaction, the AgNWs, Au nanoparticles, Fe_3_O_4_ nanospheres, and PPy nanoparticles inside the device performed as thermal sources would facilitate the interfacial fusion of the damaged parts under the external stimuli. During the heating process, the mobility of sulfur-atom-ended polymer network was greatly improved, which enabled the reconstruction of polymer chains on the metal surface from the neighboring parts for secondary combination. As schematically illustrated in Fig. 4b, the crack interfaces among the current collector/electrode/electrolyte layers of the supercapacitor can be healed owing to the interfacial reconstruction of dynamic Au, Ag–SR bonds inspired by distinct photothermal, galvano-thermal, and magneto-thermal effects under NIR laser irradiation, electric current, and alternating magnetic field, respectively.

**Fig. 5:**
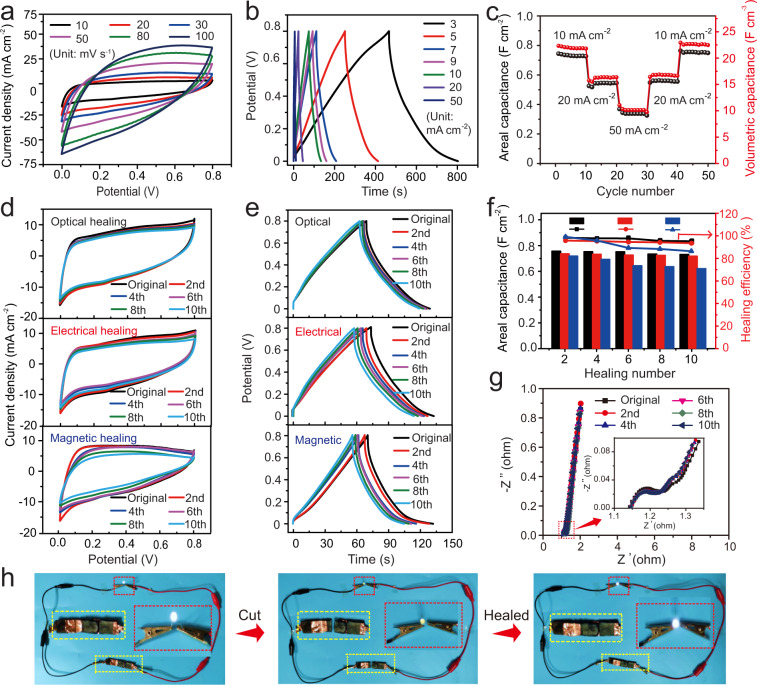
Multi-responsive healing performance of the supercapacitor. **a** CV curves at different scan rates. **b** GCD curves at different current densities. **c** Rate performance of areal and volumetric capacitances at current density ranging from 10 to 50 mA cm^−2^. Optical, electrical, and magnetic healing process over 10 cutting-healing cycles: **d** CV curves at a scan rate of 10 mV s^−1^, **e** GCD curves at a current density of 10 mA cm^−2^, **f** areal capacitance and healing efficiency during optical (black), electrical (red), and magnetic (blue) healing process, and **g** EIS spectra during the optical healing processes. The inset showing the enlarged view of the rectangular area in the high-frequency region. **h** Optical images show the lighting of a LED bulb by three supercapacitors connected in series during the cutting-healing process. The yellow and red squares are the enlarged photographs of the capacitor and bulb, respectively.

The capacitive performance of the assembled supercapacitor device was evaluated by cyclic voltammetry (CV) and galvanostatic charge-discharge (GCD) measurements using a two-electrode method. As shown in Fig. 5a, the quasi-rectangular CV profiles with the mirror-image symmetry were presented at low scan rates from 10 to 50 mV s^−1^. At a higher scan rate of 100 mV s^−1^, the diffusion limitation and the increasing transfer resistance resulted in the CV curve deviating from the rectangular shape. The surrounded CV areas dependent on the scan rates suggested ideal capacitive behaviors of the supercapacitor. Figure 5b showed the GCD curves with typical symmetric triangular shapes at the current densities from 3 to 50 mA cm^−2^ and a potential window of 0–0.8 V. It was calculated that the areal capacitance reached 1264 mF cm^−2^ at a current density of 3 mA cm^−2^, which was the highest value among the previously reported healable supercapacitors (Supplementary Table 1). Notably, the supercapacitor demonstrated excellent rate performance on areal capacitance (Fig. 5c and Supplementary Fig. 15). When increasing the current densities from 10 to 50 mA cm^−2^, the areal capacitances were stable at each rate. Even at a high current density of 50 mA cm^−2^, a capacitance of 309 mF cm^−2^ was estimated, which was greater than that of the previously reported flexible/healable supercapacitors at low current densities^5,33–35^. Since the current density was back to 10 mA cm^−2^, its capacitance was recovered to 727 mF cm^−2^. In the extended GCD tests at a current density of 10 mA cm^−2^, the device showed capacitance retention of 96.1% after 5000 charge-discharge cycles (Supplementary Fig. 16a). However, the high proportion of interconnected porous structure and moderate density of PPy enabled the assembled supercapacitor with a limited volumetric capacitance (22.3 F cm^−3^ at 10 mA cm^−2^) (Fig. 5c). To reveal the anti-drying stability of the hydrogel-based supercapacitor, the electrochemical performance of the assembled device during the drying process was systematically investigated. Firstly, the influence of dehydration of hydrogel electrolyte on the capacitance performance was studied. By real-time monitoring the water variation of the electrolyte exposed to the air at room temperature with the humidity of 40–50%, a relatively slow dehydration was indicated with ~25% of water loss in 86 h (Supplementary Fig. 17a). The capacitive performance of the assembled supercapacitor was gradually decreased with 71% of original capacitance retained correspondingly (Supplementary Fig. 17b). Benefited from the densely crosslinked network with enhanced capability of water retention, an integrated supercapacitor when exposed to the air also exhibited a slow dehydration behavior (Supplementary Fig. 18a). When the water content was decreased from the original 74% to 51% in 72 h, 64% of the specific capacitance was still retained (Supplementary Fig. 18b). In contrast, the two comparative supercapacitors assembled from the CCP/PPy and PMP/PPy electrodes delivered much weakened capacitive performances as indicated from the irregular rectangular CV shapes with the smaller areas (Supplementary Fig. 19a). Concretely, calculated from the corresponding GCD curves, the areal capacitances of 242 and 422 mF cm^−2^ were determined for the CCP/PPy and PMP/PPy supercapacitors, respectively (Supplementary Fig. 19b), much lower than 1264 mF cm^−2^ of the MFP-PPy device. As revealed in the electrochemical impedance spectroscopy (EIS) spectra (Supplementary Fig. 20), the MFP-PPy supercapacitor presented excellent electrical conductivity by delivering a low charge-transfer resistance of 0.12 Ω as estimated from the semicircle diameter in the high-frequency region. Furthermore, the straight line in a steep slope in the low-frequency region indicated a small electrolyte diffusion resistance and good capacitive behavior. Contrarily, the two reference supercapacitors exhibited much larger resistance, indicating inferior electrical conductivities (Supplementary Fig. 20). In contrast to the CCP/PPy and PMP/PPy, the employment of BACA-modified Fe_3_O_4_@Au nanocomposites as crosslinkers offered the advantage of uniform and controlled porous morphology in the MFP hydrogel. The detailed information on the internal porous morphology was investigated through mercury intrusion porosimeter (MIP) (Supplementary Fig. 13b). Compared to the CCP/PPy and PMP/PPy electrodes, the MFP-PPy hydrogel electrode presented more uniform pore structures, as indicated from the narrower pore distributions in different regions of the MIP analysis, which was in favor of electron transport and electrolyte diffusion during the electrochemical process. Based on these electrochemical analyses, the combined merits of high electrical conductivity and an integrated device configuration along with the structural advantages of the electrode and electrolyte resulted in superior performance of the MFP-PPy supercapacitor.

Further electrochemical measurements were carried out to demonstrate multi-responsive healing performance of the MFP-PPy assembled supercapacitor under the optical, electrical, and magnetic stimuli, respectively. As shown in Fig. 5d, the device presented the nearly overlapped regular-shaped CV curves before and after different cutting-healing cycles at a scan rate of 10 mV s^−1^, indicating its non-deteriorated capacitive performances under three external stimuli, respectively. Moreover, the GCD curves of the device were highly consistent in both shape and discharging time before and after healing cycles at a current density of 10 mA cm^−2^, suggesting no obvious degradation in capacitance (Fig. 5e). In comparison with magnetic and electric healing, the optically healed device presented the lowest deviation on the GCD curves arising from the highest healing efficiency of NIR laser irradiation with the strongest heating capability (Supplementary Fig 21a). In addition, 86.3% of mechanical healing efficiency in 2 min proved the highly efficient healing performance under the stimulus of NIR (Supplementary Fig. 21b). For clearness, Fig. 5f showed the areal capacitances and healing efficiencies of the device during the cutting-healing cycles under different stimuli calculated from the GCD curves. After the 10th optical, electrical and magnetic healing processes, the supercapacitor restored the areal capacitance of 710, 702, and 609 mF cm^−2^ at the current density of 10 mA cm^−2^, which was estimated to be 95.2%, 93.8%, and 86.1% in healing efficiency, respectively. Compared with NIR laser-induced healing, the smaller capacitance retention was demonstrated during the magnetic healing process, which was resulted from moderate magnetic heating capability under the mild magnetic field, as confirmed by 52% of mechanical healing efficiency in 10 min (Supplementary Fig. 21b). Considering the healing mechanism of heating-induced metal-coordination interface reconstruction, the heating capability of the Au surface in the gel determined the healing efficiency. For the optical healing process, the heating was directly produced from the Au nanoparticles with the notable photothermal property, resulting in a rapid and highly efficient healing. In the magnetic healing process, the heating of the Au surface was indirect, which was transferred from the neighboring Fe_3_O_4_ spheres. Moreover, unsatisfactory thermal-conduction of Fe_3_O_4_ led to heat loss inevitably. Herein, a bit deteriorated electrical healing efficiency was because of unavoidable thermal loss in the heat transfer between PPy and Au nanoparticles, and continuous swelling and shrinking of the polymer during the charge–discharge process for the electrical healing^36^.

EIS spectra of the device after different healing cycles coincided with the original profile, suggesting good restorations of the electrical conductivity during the stimulus-inspired healing process (Fig. 5g and Supplementary Fig. 22). The small impedance arcs in the high-frequency region and small intercept along Z′ indicated low charge-transfer resistance between the electrolyte and electrode and equivalent series resistance of the healed devices. The nearly vertical profile for the healed supercapacitors in the low-frequency region revealed an ideal capacitive behavior. SEM images showed that the broken interface of the device was glued together and presented a well-defined interconnected network, confirming its effective healing behavior (Supplementary Fig. 23). The optical images exhibited the stable brightness of the light-emitting diode (LED) bulbs even when the healed device was stretched at high strains (Supplementary Fig. 24), revealing a highly efficient healing of the MFP-PPy supercapacitor. Furthermore, the healed supercapacitor showed excellent long-term cycling stability, as proved by 93.4% of capacitance retention after 5000 GCD cycles at a current density of 10 mA cm^−2^ (Supplementary Fig. 16b). Consequently, the supercapacitors could power the LED bulb after the cutting-healing operation (Fig. 5h).

## Discussion

As compared with previously reported self-healable supercapacitors, several advantages can be easily found and summarized. First, the healable layers including both electrodes and electrolyte were combined through the healing-induced strategy under external stimuli for all-solid-state flexible and stretchable supercapacitors^37^. This integrated configuration containing reversible interfaces with rich metal-thiolate interaction between current collectors, hydrogel electrodes, and electrolyte layer, enables the device with excellent capacitance performance as well as great stretchability and healing behaviors. Especially, the exclusive interfacial interaction of metal-thiolate coordination throughout the devices facilitates the healing performance in a rapid and highly efficient way within single stimulus (Supplementary Table 1). Second, the fabricated supercapacitors show great promise as power sources in case of being integrated with other active devices for realization of wearable electronics since their professional flexibility and stretchability^38,39^. In contrast to other healable supercapacitors with single-stimulus responsive property reported, the multi-stimulus induced self-healing performance of our fabricated supercapacitors would provide additional advantages when the neighboring active devices are also stimulus-responsive, for example, sensors. Depending on the components of the active devices, appropriate stimulus can be chosen and performed to heal the damaged region, accompanied with little triggering effect on the neighboring devices. As such, the fabricated supercapacitors have been demonstrated to be self-healable under various external stimuli including NIR light, electricity, and alternating magnetic field. Based on the device integration, the healing experiments can be carried out independently, far away from the active device. It is noteworthy that the stimuli of light and magnet can be performed in a remote and located way, which can further decrease the undesirable responsiveness. Last but not the least, these synthetic materials with professional mechanical and self-healing performances would present great significance to the sustainable development and environmental protection. Despite higher cost than the biomass-based counterparts^40–42^, the synthetic materials could provide great advantages on the structural design and interfacial control for the purpose of professional mechanical and functional properties, such as mechanical strength, stretchability, and self-healing behavior. Specially, the excellent stretchable performance enables the integrated device with broad applications in versatile environments under multiple mechanical deformations. The realization of materials with self-healing capability, as an efficient and cost-effective alternative, can increase the lifetime of the structures and decrease their maintenance cost. Such an intrinsically healable design guaranteed the integrated structural configuration and good recovery in electrical conductivity and mechanical property, and therefore promised high capacitive performance and healing efficiency of the device, confirming the MFP hydrogel-assembled supercapacitor as one of the best performers among the flexible and healable supercapacitors.

In conclusion, we have demonstrated an optically, electrically, and magnetically responsive self-healing supercapacitor device with the integrated configuration soldered by dynamic Ag, Au–SR bonds assembled from the MFP-based hydrogel electrodes and electrolyte and AgNW films as current collectors. Resulting from the chemically crosslinked Fe_3_O_4_@Au nanocomposite in the polymer network, the MFP hydrogel exhibited tough mechanical strength with a high strechability of 2250% and notable notch-insensitivity. Furthermore, its notable photothermal and magneto-thermal properties enabled the hydrogel with fast optical and remotely controllable magnetic healing relied on high-density dynamic Au–SR bonds. With the merits of the porous structure, strong mechanical property and intrinsic healability of the electrode and electrolyte, the assembled supercapacitor delivered a largest areal capacitance of 1264 mF cm^−2^ among the reported healable supercapacitors and superhigh device-level stretchability of 1200%. As a demonstration of triply-responsive self-healing supercapacitor, the device presented excellent healing performance with ~90% of capacitances restored over ten optical, electrical, and magnetic healing cycles, respectively. This intrinsic self-healing integration strategy along with the advanced electrode and electrolyte proposed here allows for assembling multifunctional and high-performance supercapacitor devices under precise interfacial controls, promising great potentials in powering next-generation wearable and portable electronic devices.

## Methods

### Preparation of Fe_3_O_4_@Au nanocomposites

In a typical synthesis, Fe_3_O_4_ nanospheres were firstly synthesized by a solvothermal method. In all, 1.8 g of FeCl_3_· 6H_2_O was dissolved in 40 mL of ethylene glycol at room temperature to form a clear solution. Then, 0.27 g of trisodium citrate, 1 g of polyvinyl pyrrolidone, and 2.4 g of sodium acetate were successively added into above solution. After stirring for 2 h, the obtained mixture was transferred to a sealed Teflon-lined stainless-steel autoclave with a capacity of 50 mL and heated at 200 °C for 10 h. After cooling to room temperature, the products were washed by water and ethanol three times with the assistance of a magnet. In the following, in situ reduction of Au nanoparticles onto Fe_3_O_4_ nanospheres was carried out. Typically, 2 mg mL^−1^ of Fe_3_O_4_ nanospheres were dispersed into the solution of ethanol and ammonia under ultrasonication. Then, 300 μL of HAuCl_4_ (1 wt%) was added into the above dispersion quickly. With stirring for 2 h, the Fe_3_O_4_@Au composites were collected by a magnet, cleaned with water and ethanol three times and dried under vacuum at 60 °C for use.

### Preparation of MFP hydrogel

In a typical procedure, the as-prepared Fe_3_O_4_@Au nanocomposites were homogeneously dispersed in 5 mL of aqueous solution. Then, the desired amount of BACA was added to the above solution to obtain the BACA-modified Fe_3_O_4_@Au composites as the multifunctional crosslinkers for the subsequent polymerization. Next, 1.0 g of AAm, 0.02 g of KPS, and 20 μL of TEMED were added with ultrasonication until all reagents were dissolved. Then, N_2_ bubbling was conducted to remove the dissolved O_2_. After that, the mixture was degassed in a vacuum drying oven. With the polymerization at room temperature for 24 h, the magnetic composite hydrogel was obtained. Similarly, several magnetic hydrogels were fabricated by using different contents of Fe_3_O_4_@Au composites, that was 0.4, 1.0, 2.0, and 4.0 mg mL^−1^, denoted as MFP-*n* (*n* = 1 – 4). Furthermore, the reference sample, physically-incorporated magnetic PAM hydrogel was also prepared by using the organic crosslinker, MBAA, instead of BACA, denoted as PMP hydrogel. In this case, Fe_3_O_4_@Au composites were not chemically crosslinked in the network because of no chemical interactions between MBAA and Fe_3_O_4_@Au composites. The conventional chemically crosslinked polymeric hydrogel was also prepared as the other control sample by using BACA as chemical crosslinker without the addition of Fe_3_O_4_@Au composites, denoted as CCP hydrogel.

### Preparation of MFP-PPy hydrogel electrode

The MFP hydrogel was firstly dehydrated at room temperature for 24 h to remove part of the water from the gel. Then, the hydrogel piece was soaked in 0.5 mol L^−1^ of pyrrole solution for 6 h to achieve the swelling equilibrium. The swollen hydrogel was placed into the yellow FeCl_3_ solution (1.0 mol L^−1^). After the polymerization at room temperature for 12 h, the MFP-PPy hydrogel electrode was prepared with washing to remove the impurities on the surface. The mass loading and corresponding mass fraction of the PPy on the MFP hydrogel were 18.2 ± 3 mg cm^−3^ and ~5 ± 1% (dry sample), respectively.

### Healing property measurements

The optical-induced healing process was executed using a NIR laser (MDL-III-808) with the wavelength of 808 nm and the power of 1.5 W. The hydrogels were cut into two pieces by a sharp blade. With the separated parts put into contact, the laser was exposed on the joint of hydrogels at a distance of 10 cm with the spot area of 1 cm × 0.5 cm at room temperature. The magnetic triggered healing process was carried out on a gel cylinder with a radius incision by a sharp blade. The fractured sample was healed when placed into the helical coil of a home-made alternating magnetic field generator at 315 KHz, 25 kA m^−1^. The exact healing time including the heating and cooling time for the MFP hydrogel-based supercapacitors was 2 min, 6 min, and 10 min in the external stimuli of NIR light, electric current, and alternating magnetic field, respectively.

### Assembly and electrochemical measurements of healable supercapacitor device

The supercapacitor device was assembled by sandwiching two pieces of MFP-PPy hydrogel electrodes (1 mm thick) with MFP hydrogel electrolyte (1 mm thick) and spray-coating AgNW films on the electrodes as current collectors. Placing at a helical coil of the alternating magnetic field, the layers of current collector, electrode and electrolyte were chemically soldered together. Prior to device assembly, the electrode and electrolyte hydrogels were soaked in 1.0 mol L^−1^ of Na_2_SO_4_ aqueous solution for 2 h. To improve the capacitive performance, the thickness of the device was reduced to be 1 mm by the compression between two metal plates. All the electrochemical tests including CV, GCD curves and EIS spectra were performed using two-electrode system on a CHI 760E electrochemical workstation at room temperature. CV curves were recorded at various scan rates from 10 to 100 mV s^−1^ and the potential range of 0–0.8 V. GCD tests were carried out at the current densities from 3 to 50 mA cm^−2^ with the potential range of 0–0.8 V. EIS spectra were tested in the frequency range of 10 mHz–100 kHz. The areal capacitance was calculated based on the equation:

$${{C}}=\frac{{{I}}\times \Delta {{t}}}{S\times \Delta {{U}}},$$

where *I* is the discharge current, Δ*t* is the discharge time and Δ*U* is the voltage window, and *S* is the area of the electrode material. The volumetric capacitance was calculated based on the equation:

$${{C}}=\frac{{{I}}\times \Delta {{t}}}{{{V}}\times \Delta {{U}}},$$

where *V* is the volume of the electrode material. The optical and magnetic healing of the damaged device was carried out with the help of NIR laser irradiation and alternating magnetic field generator, respectively. The real-time electrical healing of the damaged device was performed through the charge–discharge cycles at a current of 50 mA. For healing cycles, the devices were cut at the same position.

### Materials characterization

SEM images were characterized by a field-emission scanning electron microscopy (Zeiss, Merlin Compact) at an acceleration voltage of 5 kV. High-resolution TEM images and the elemental mapping profiles were carried out on a JEM-2100F field-emission transmission electron microscopy equipped with an Oxford Inca energy instrument at 200 kV. XPS were recorded on an ESCALab MKII X-ray photoelectron spectrometer by means of a monochromatic X-ray source. XRD patterns were recorded on a Philips X’Pert PRO MPD X-ray diffractometer by using Cu Ka radiation. MIP study was performed on an AutoPore IV 9510 for detailed analysis of the pore morphology of hydrogels. All the mechanical curves were recorded on the Instron 5965A mechanical testing system with a 100 N load cell. The tensile experiments were conducted on the hydrogel pieces with a length of 15 mm, width of 5 mm, and thickness of 2 mm at a stretch rate of 10 mm min^−1^. Infrared thermal images were conducted on a Fluke Ti400 infrared imager.

## Supplementary information

Supplementary Information

## Data Availability

All data generated or analyzed during this study are included in the published article and its Supplementary Information. Raw data are available on reasonable request from the corresponding authors (H.-P.C. or S.-H.Y.).
