# Supplementary material for: A multi-responsive healable supercapacitor
Source: Nat Commun. 2021 Jul 14;12:4297. doi: 10.1038/s41467-021-24568-w (PMC8280176; doi:10.1038/s41467-021-24568-w)
Supplement: Supplementary file 1 — Supplementary Information [file 41467_2021_24568_MOESM1_ESM.pdf]

Supplementary Information

A multi-responsive healable supercapacitor

Haili Qin[1], Ping Liu[1], Chuanrui Chen[1], Huai-Ping Cong[1]*, Shu-Hong Yu[2]*

[1] Anhui Province Key Laboratory of Advanced Catalytic Materials and Reaction Engineering, School of Chemistry and Chemical Engineering, Hefei University of Technology, 230009, P. R. China

[2]Division of Nanomaterials and Chemistry, Hefei National Laboratory for Physical Sciences at Microscale, Institute of Energy, Hefei Comprehensive National Science Center, Department of Chemistry, Institute of Biomimetic Materials & Chemistry, University of Science and Technology of China, 230026, P. R. China

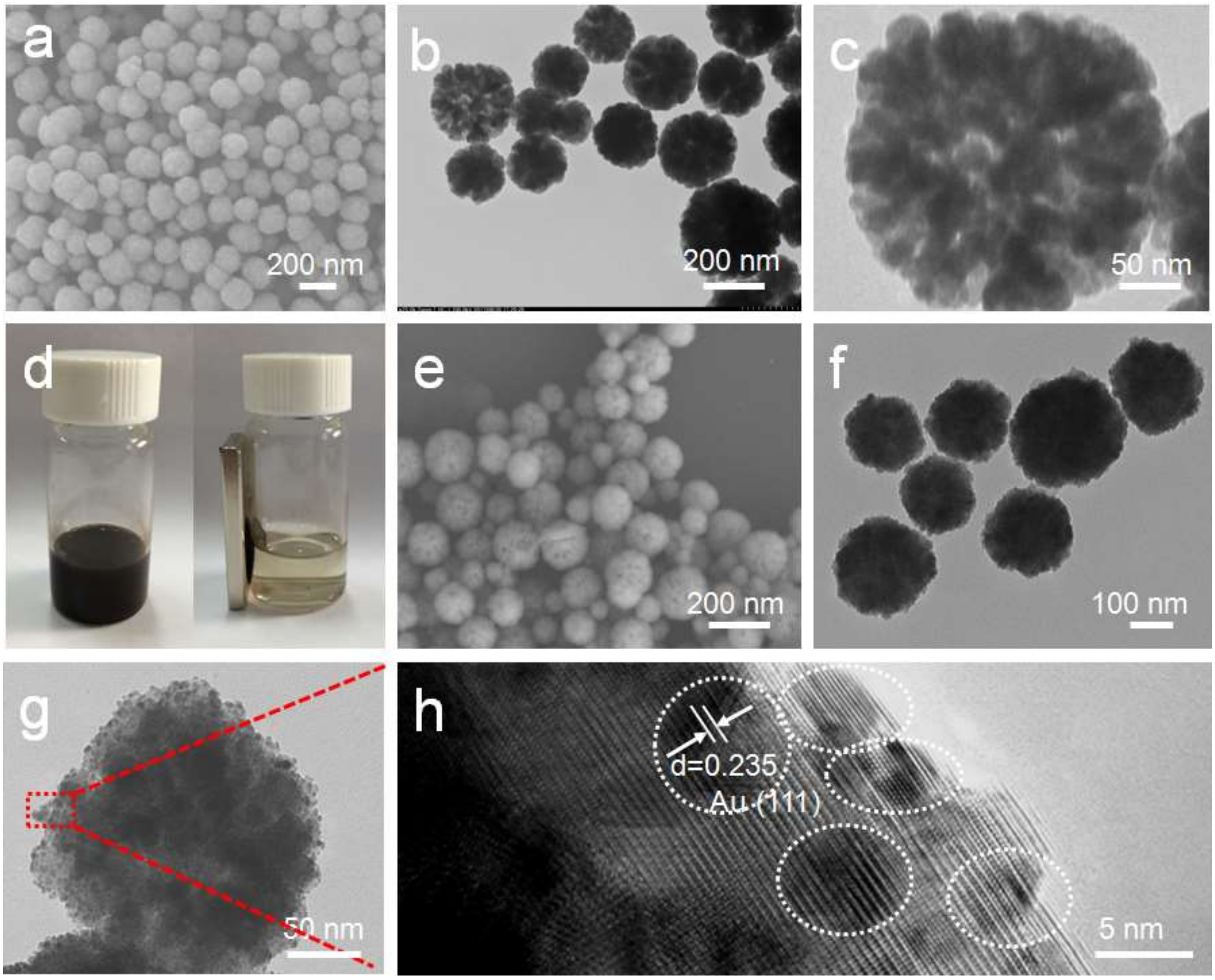

Supplementary Figure 1. (a) SEM image of Fe_3O_4 nanospheres. (b, c) Different magnifications of TEM images of Fe_3O_4 nanospheres. (d) Optical images show magnetic response of Fe_3O_4 aqueous dispersion with a magnet. (e) SEM image of Fe_3O_4@Au nanocomposites. (f, g) Different magnifications of TEM images of Fe_3O_4@Au nanocomposites. (h) HRTEM image of Fe_3O_4@Au nanocomposite in the squared area of (g).

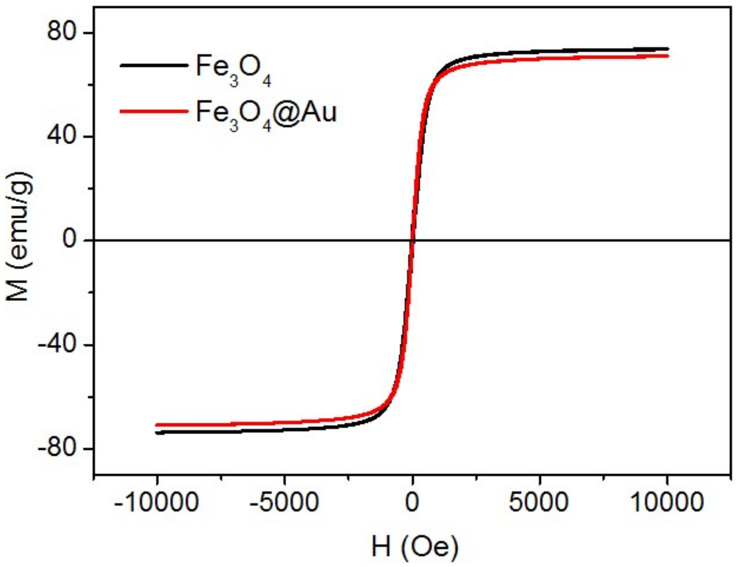

Supplementary Figure 2. Magnetic hysteresis loops of Fe_3O_4 nanospheres and Fe_3O_4@Au nanocomposites.

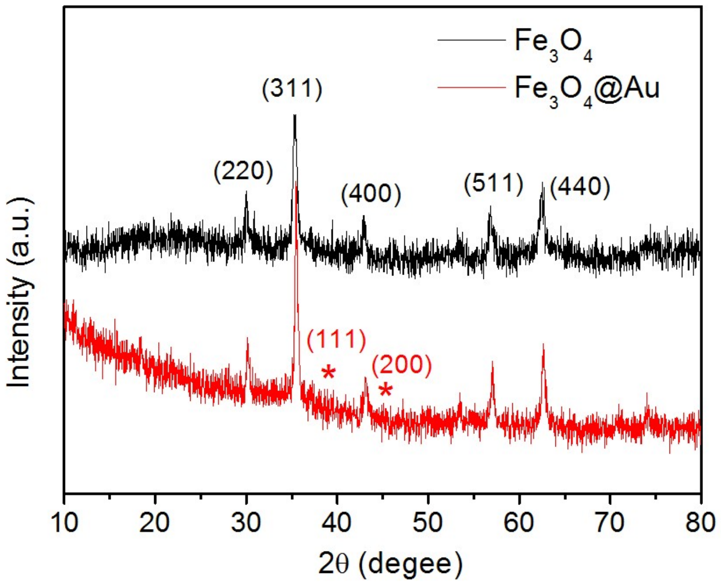

Supplementary Figure 3. XRD patterns of Fe_3O_4 nanospheres and Fe_3O_4@Au nanocomposites.

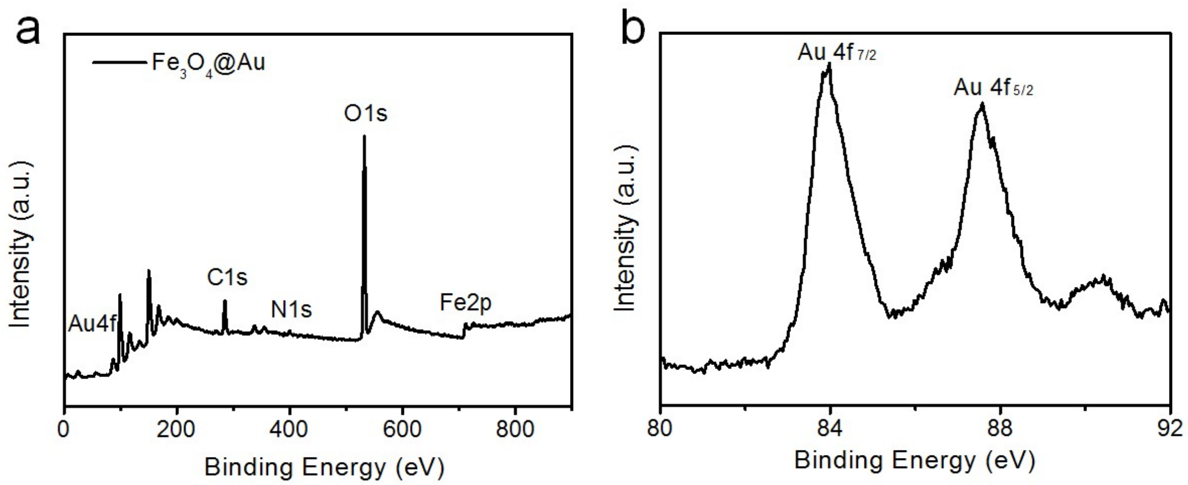

Supplementary Figure 4. (a) Survey XPS spectrum of Fe_3O_4@Au nanocomposites. (b) Core-leveled Au 4*f* XPS spectrum of Fe_3O_4@Au nanocomposites.

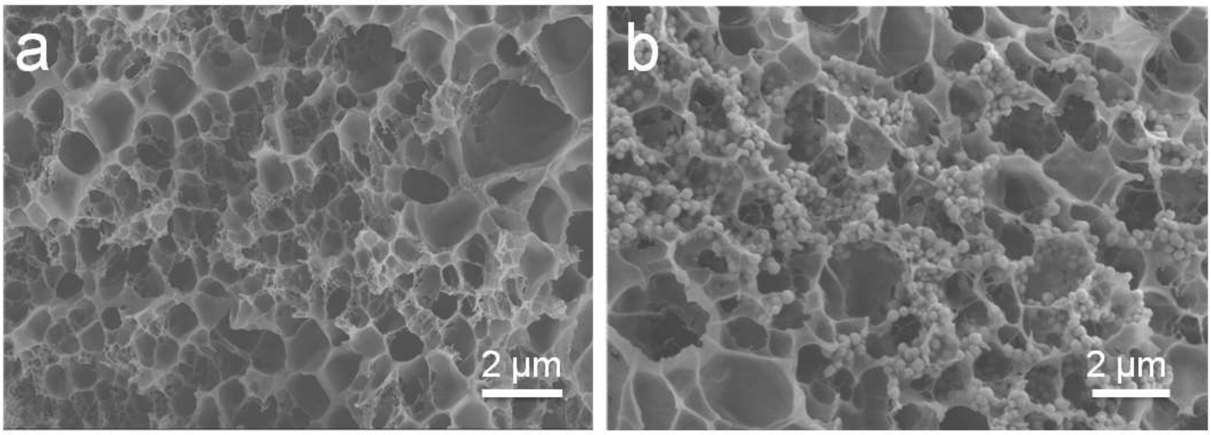

Supplementary Figure 5. SEM images of (a) CCP hydrogel and (b) PMP hydrogel.

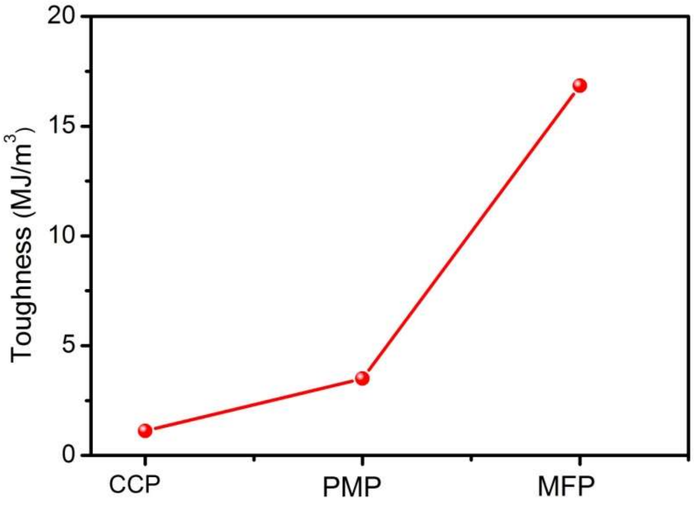

Supplementary Figure 6. Toughness of CCP, PMP and MFP hydrogels.

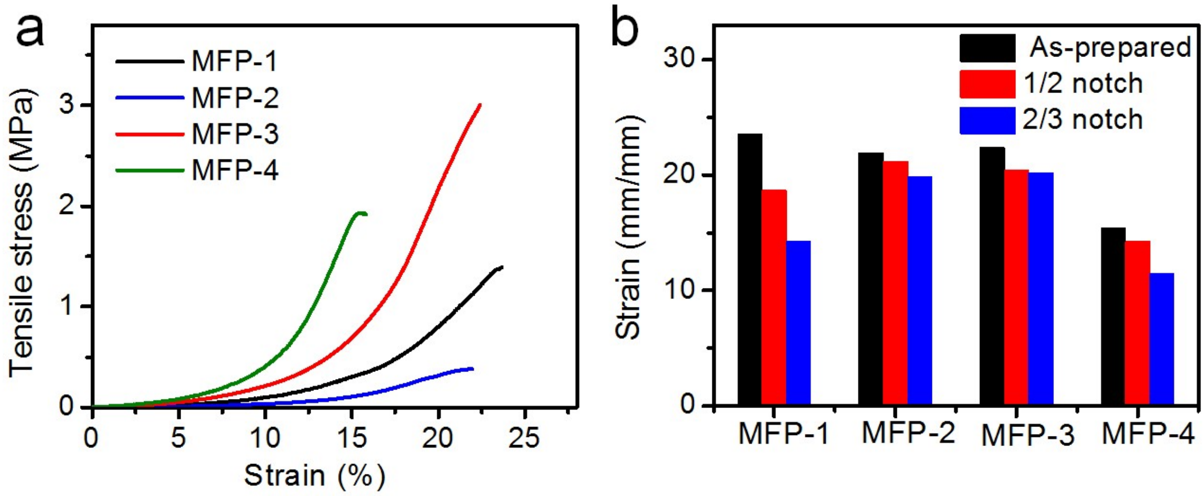

Supplementary Figure 7. (a) Tensile stress-strain curves of MFP hydrogels with different contents of Fe_3O_4@Au nanocomposites. (b) Strains of MFP hydrogels notched with different sizes. The content of Fe_3O_4@Au is 0.4, 1.0, 2.0 and 4.0 mg mL^{-1}, respectively.

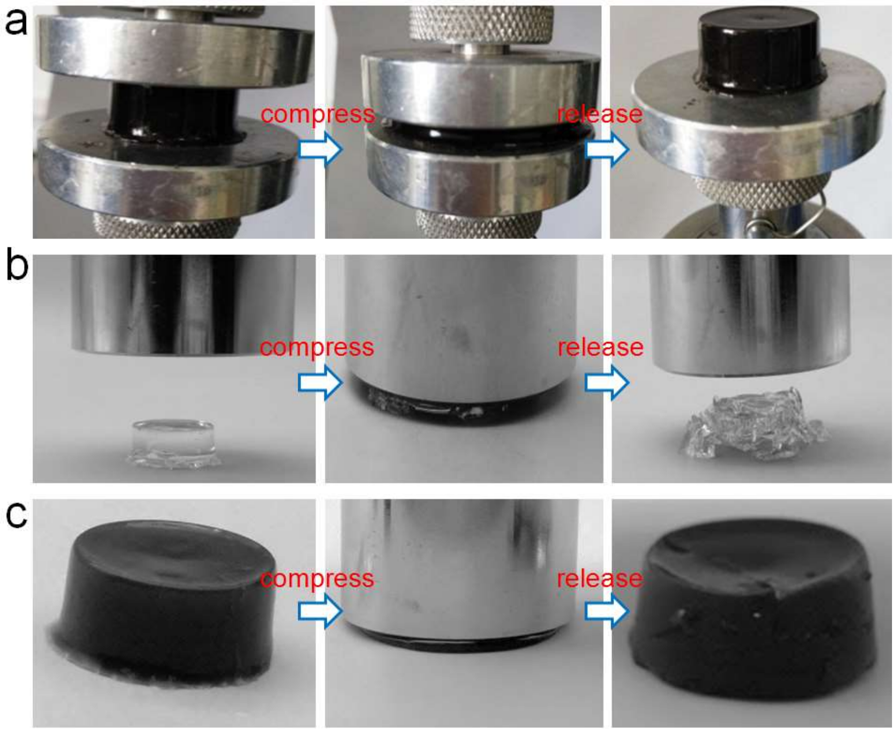

Supplementary Figure 8. Optical images of the shape changes of (a) MFP hydrogel, (b) CCP hydrogel and (c) PMP hydrogel during the compress-release process.

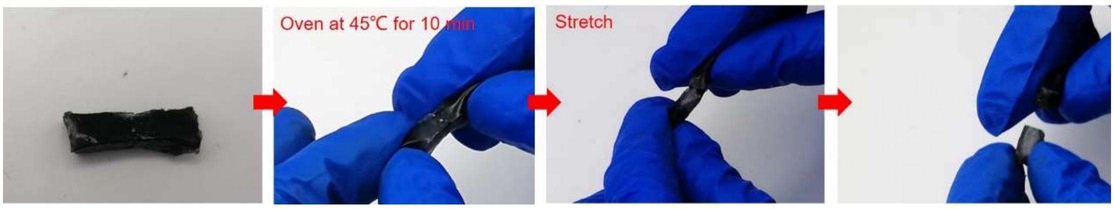

Supplementary Figure 9. Optical images for the self-healing experiment of MFP hydrogels heated in the oven as the thermal stimulus.

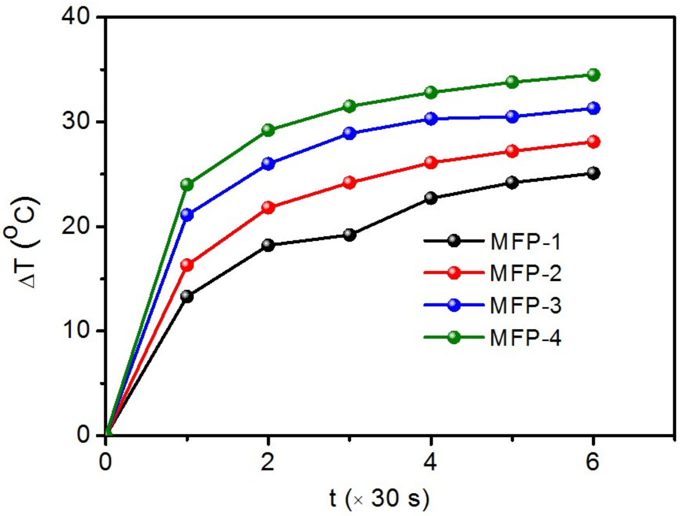

Supplementary Figure 10. Temperature change of MFP hydrogels with different contents of Fe_3O_4@Au nanocomposites during the irradiation of NIR laser (content: 0.4, 1.0, 2.0 and 4.0 mg mL^{-1}).

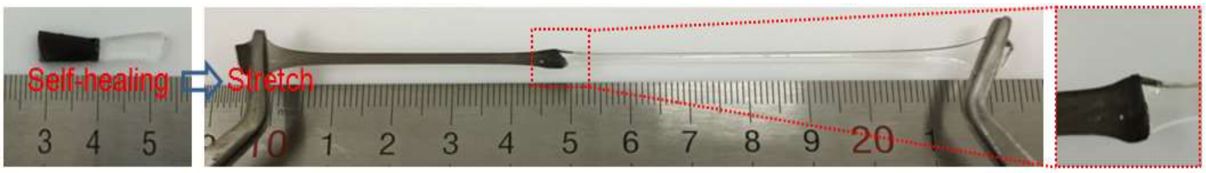

Supplementary Figure 11. Optical image of high stretchability of the healed hydrogel pieces consisting of MFP hydrogel and CCP hydrogel.

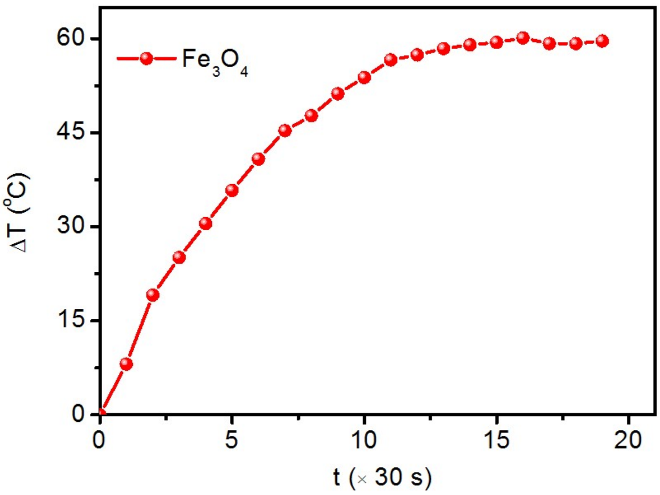

Supplementary Figure 12. Time-dependent temperature change of Fe_3O_4 nanospheres under the alternating magnetic field.

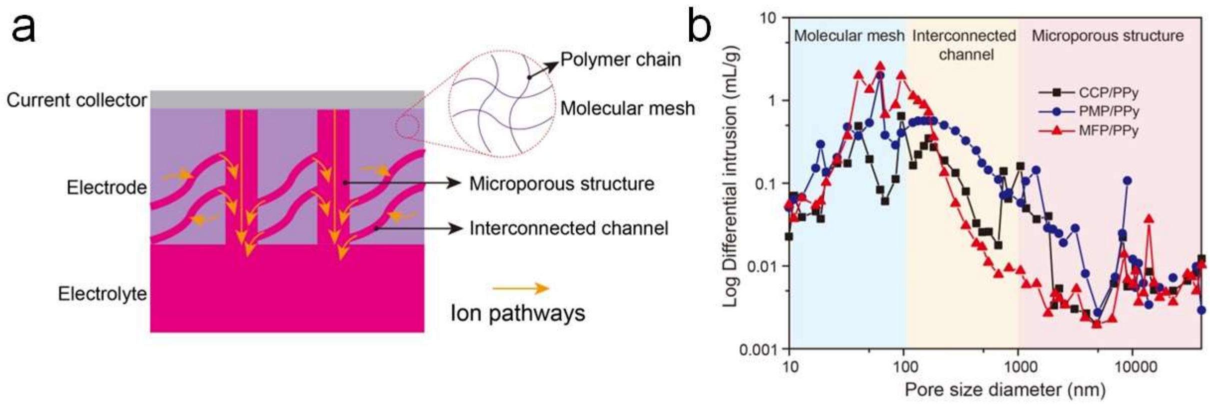

Supplementary Figure 13. (a) Schematic illustrating the ion transfer pathway in the hydrogel electrode. (b) Detailed analysis of pore structure for CCP/PPy, PMP/PPy and MFP/PPy hydrogel electrodes using MIP.

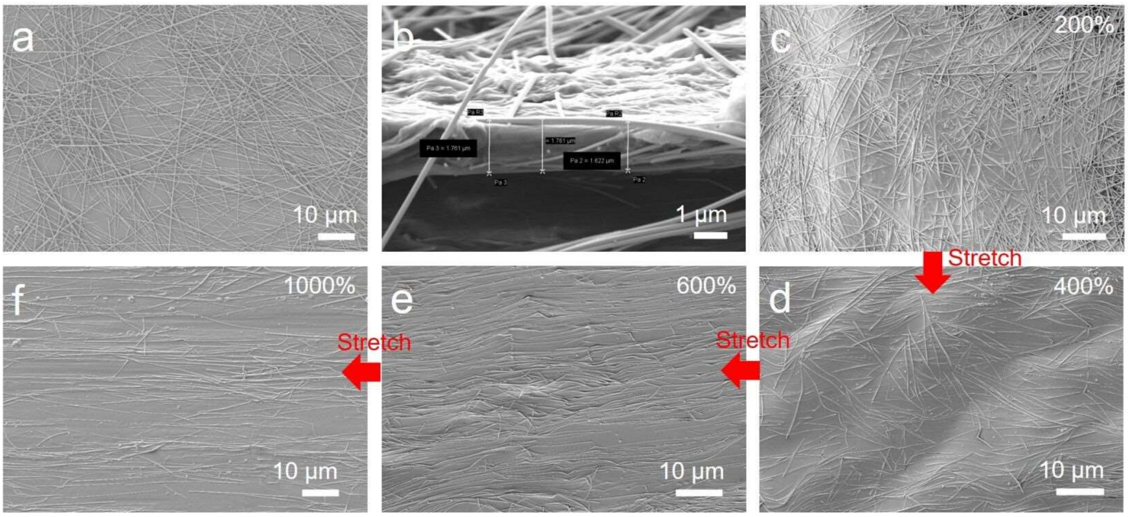

Supplementary Figure 14. (a) SEM image of AgNWs. (b) Cross-sectional SEM image of the current collector. (c-f) SEM images of the current collector during the continuous stretching process at strains from 200 % to 1000%.

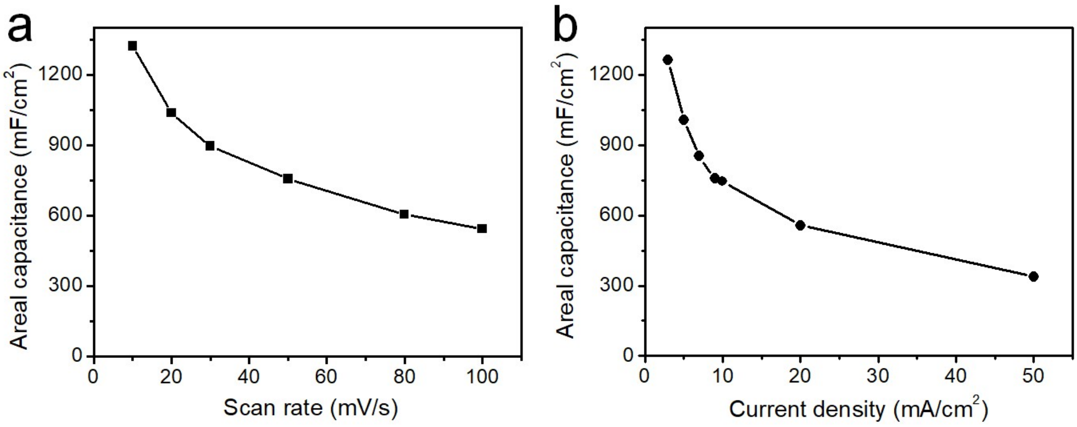

Supplementary Figure 15. Areal capacitance of the supercapacitor as a function of scan rate (a) and current density (b) calculated from CV and GCD curves, respectively.

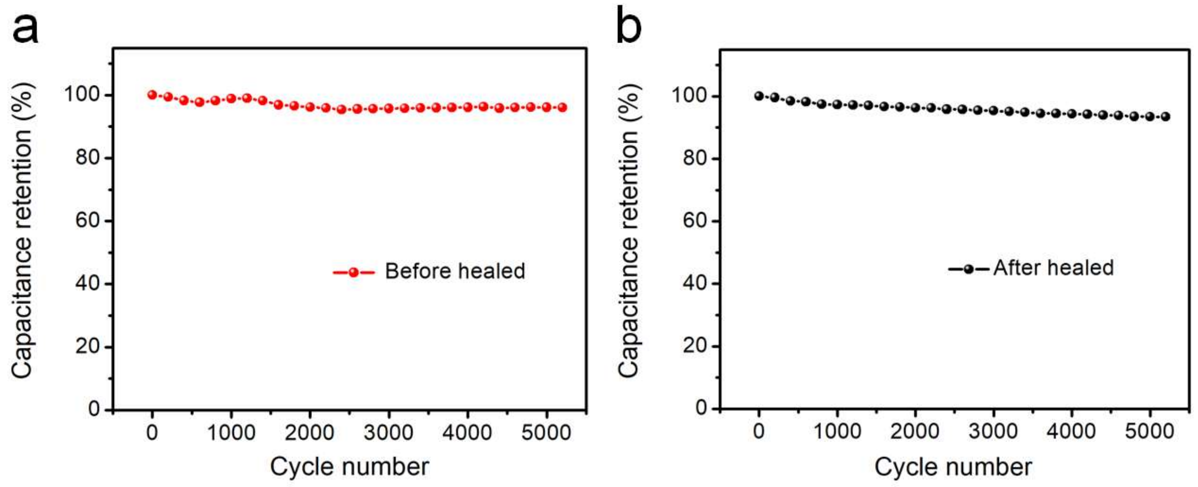

Supplementary Figure 16. Cycling performance of the supercapacitor before (a) and after (b) healing based on GCD curves at a current density of 10 mA cm^{-2}.

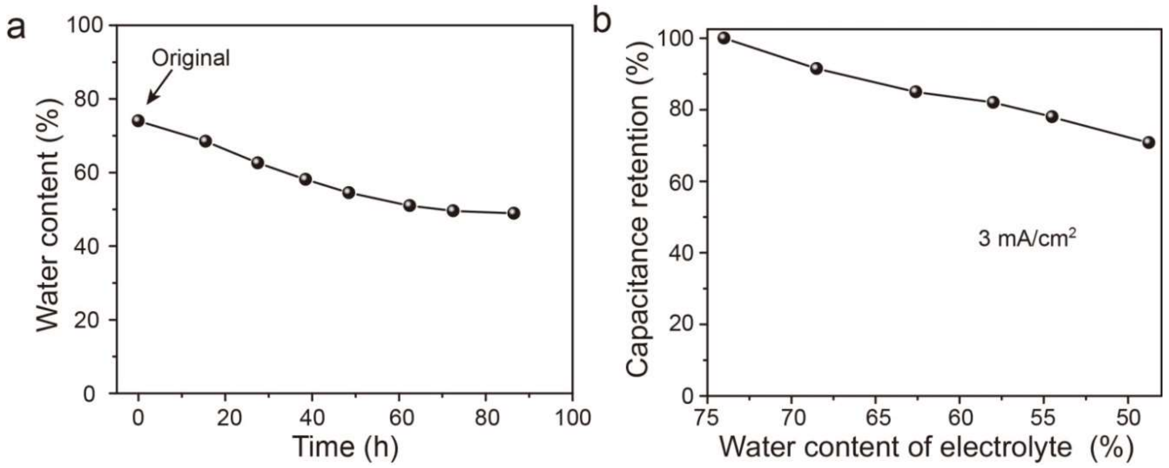

Supplementary Figure 17. (a) Monitoring the water content of hydrogel electrolyte when exposed to the air (ambient humidity: 40 ~ 50%). (b) Capacitance investigation on the dehydration effect of the MFP hydrogel electrolyte when employed for assembly of supercapacitors.

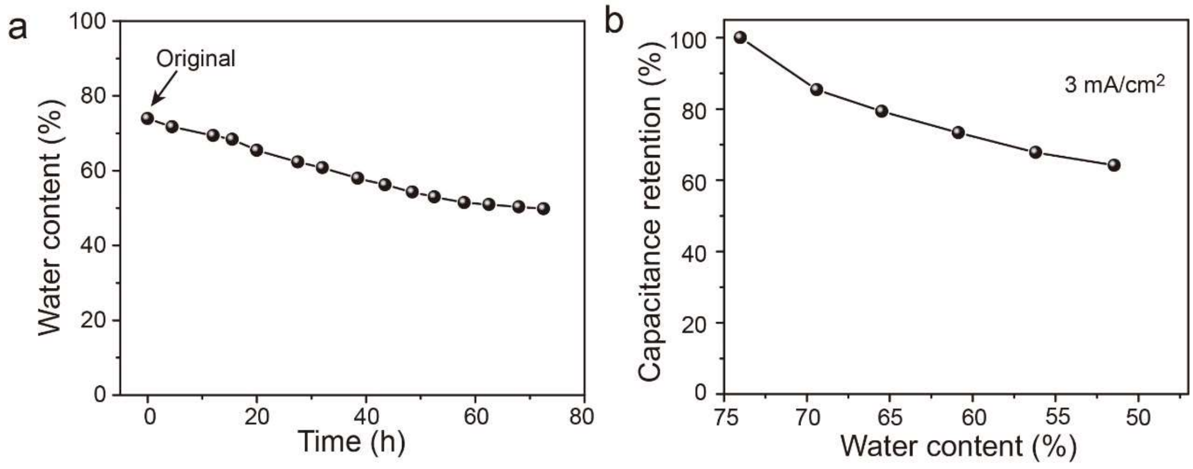

Supplementary Figure 18. (a) Monitoring the water content of hydrogel-based supercapacitor when exposed to the air (ambient humidity: 40~50 %). (b) Capacitance retention derived from GCD curves of the device with varying water content.

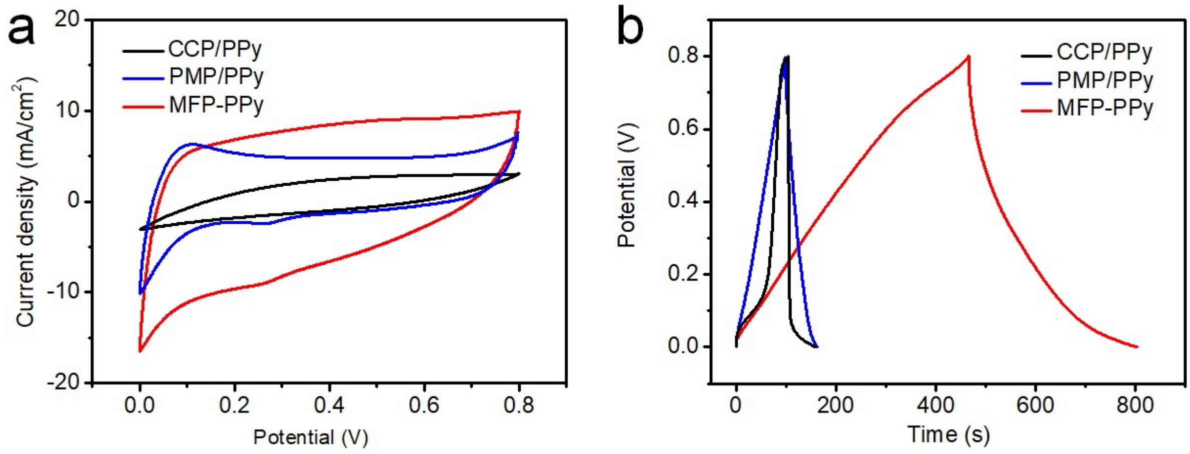

Supplementary Figure 19. (a) CV curves at scan rate of 10 mV s^{-1} and (b) GDC curves at current density of 3 mA cm^{-2} of the supercapacitor devices assembled from CCP/PPy, PMP/PPy and MFP-PPy electrode, respectively.

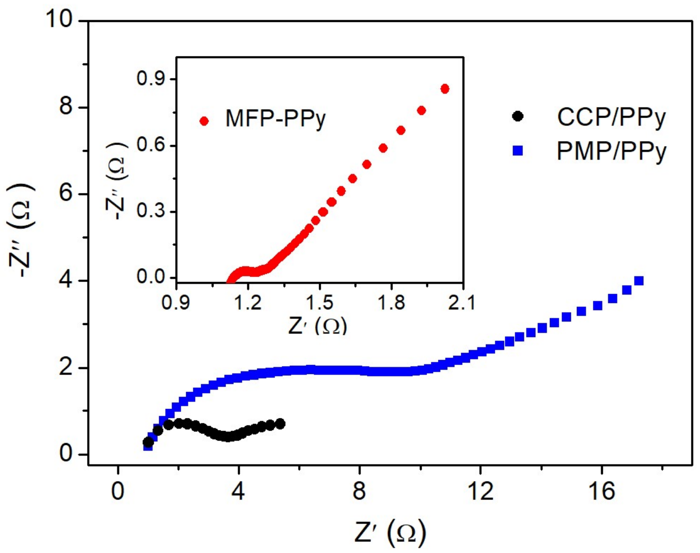

Supplementary Figure 20. EIS spectra of the supercapacitor devices assembled from CCP/PPy, PMP/PPy and MFP-PPy electrode, respectively.

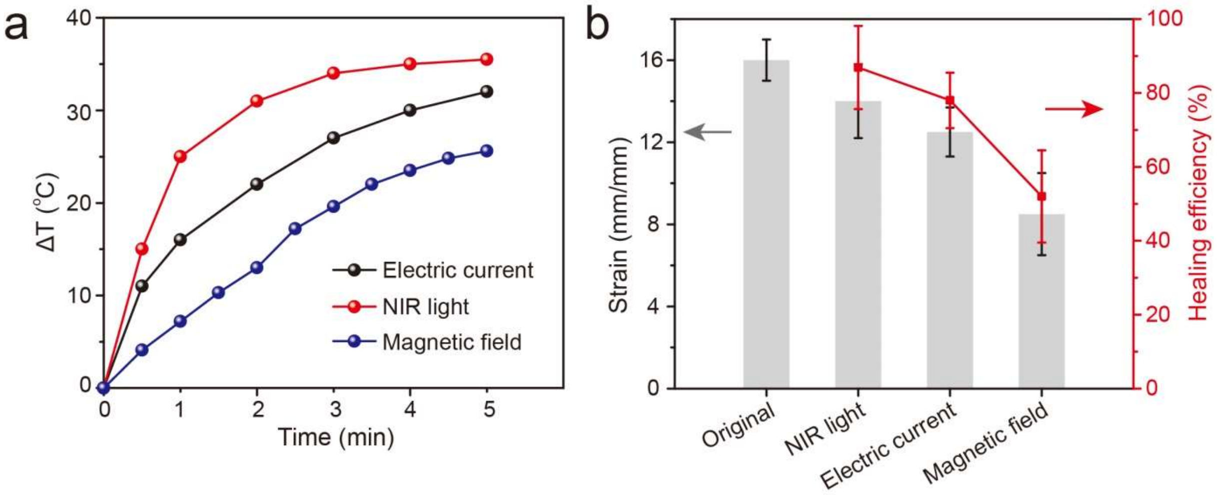

Supplementary Figure 21. (a) Temperature changes of the MFP-PPy electrodes under the stimuli of NIR laser, electric current and magnetic field. (b) Strains and healing efficiency of original and healed electrodes under different stimuli. Error bars show the SD with sample size of 3.

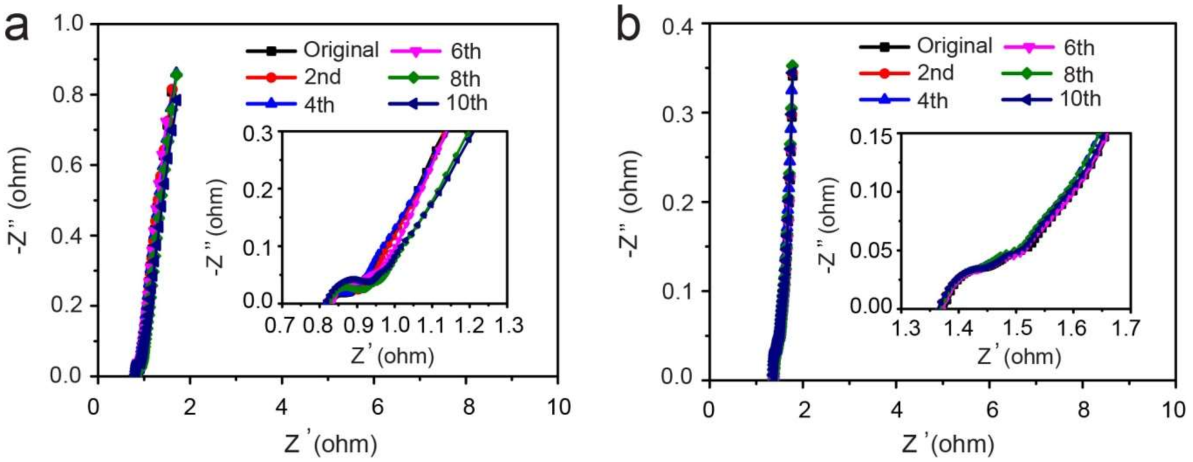

Supplementary Figure 22. EIS spectra of the supercapacitor during the electrical (a) and magnetic (b) healing processes over ten cutting-healing cycles. The inset showing the EIS spectra in the high-frequency region.

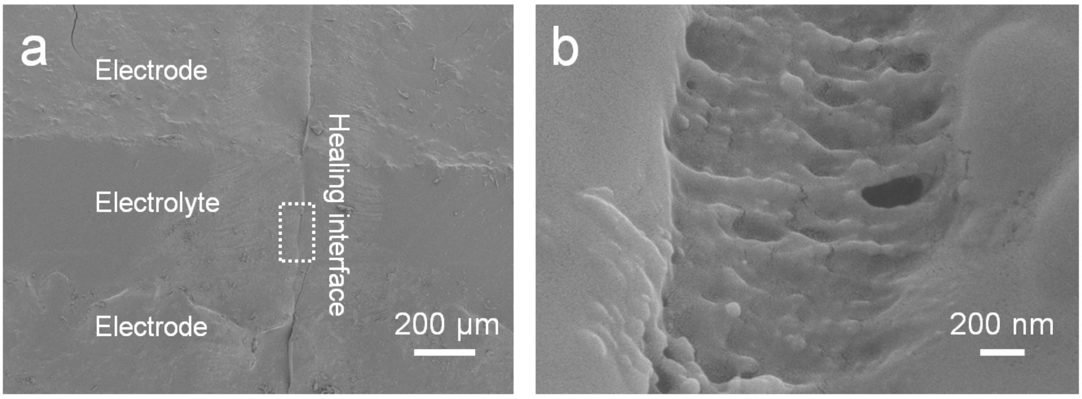

Supplementary Figure 23. SEM image (a) and enlarged SEM image (b) of the healed interface of the supercapacitor under alternating magnetic field.

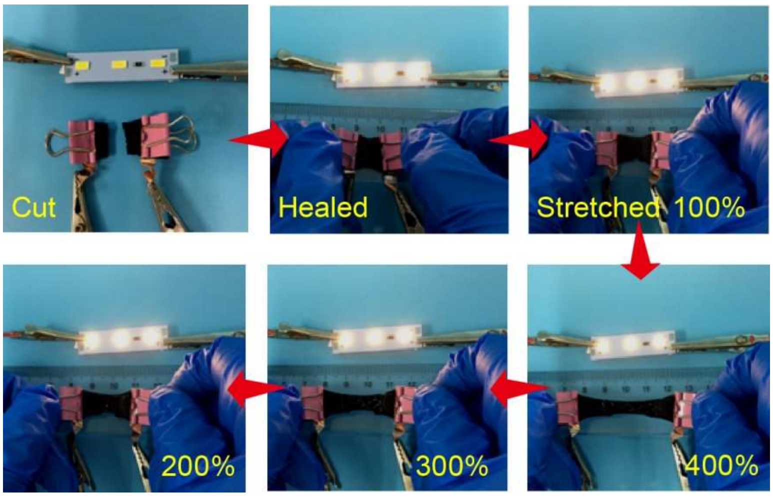

Supplementary Figure 24. Optical images show high electrical conductivity of the healed supercapacitor at large stretching deformations.

Supplementary Table 1. Comparison of the self-healing performance of the MFP-based supercapacitor with the previously-reported self-healable supercapacitors.

**Electrode**	**Electrolyte**	**Healing mechanism**	**Healable layer**	**Integrated configuration (Y/N)**	**Multi-responsiveness (Y/N)**	**Specific capacitance/ Current density**	**Ref.**
SWCNT films	PVP-H_2SO_4	Self-healable substrate	Electrode	N	N	35 F g^{-1} (1 A g^{-1})	1
PPy/Fe_3O_4 /yarn	PVA-H_3PO_4	Self-healable PU	Electrode	N	N	61.4 mF cm^{-2} (10 mV s^{-1})	2
CNT/Ag NW/CNT network	PVA-H_2SO_4	Self-healable polymer core	Electrode	N	N	140.0 F g^{-1} (0.33 A g^{-1})	3
PPy/RGO/ CNT	PVA-H_3PO_4	Healable PU	Electrode	N	N	--	4
SWCNT/PANI	PVA-H_2SO_4	Hydrogen bond	Electrolyte	N	N	15.8 mF cm^{-2} (0.044 mA cm^{-2})	5
Graphene foam@PPy	Fe^{3+}/PAA-KCl	Hydrogen bonding/ionic bond	Electrolyte	N	N	90.3 F g^{-1} (0.5 A g^{-1})	6
Activated carbon	SA-*g*-DA/KCl	Catechol-borate ester bond	Electrolyte	N	N	97 F g^{-1} (1 A g^{-1})	7
Activated carbon	PVA-*g*-PAA/ KCl	Diol-borate ester bond	Electrolyte	N	N	85.4 F g^{-1} (1 A g^{-1})	8
PPy@CNT paper	VSNPs-PAA	Healable electrolyte	Electrolyte	N	N	--	9
Activated carbon	PVA-*g*-TMAC/KCl	Diol-borate ester bond	Omni	Y	N	89 F g^{-1} (1 A g^{-1})	10
GCP@PPy	GP	Metal-thiolate bond	Omni	Y	Y (Optical/ electrical healing)	885 mF cm^{-2} (1 mA cm^{-2})	11 (Our previous work)
**MFP-PPy hydrogel**	**MFP hydrogel**	**Ag, Au-RS bond**	**Omni**	**Y**	**Y (Optical/ electrical/ magnetic healing)**	**1264 mF cm^{-2} (3 mA cm^{-2})**	**This work**

Supplementary References

1. Wang, H. *et al.* A mechanically and electrically self-healing supercapacitor. *Adv. Mater.* **26**, 3638-3643 (2014).
2. Huang, Y. *et al.* Magnetic-assisted, self-healable, yarn-based supercapacitor. *ACS Nano* **9**, 6242-6251 (2015).
3. Sun, H. *et al.* Self-healable electrically conducting wires for wearable microelectronics. *Angew. Chem. Int. Ed.* **53**, 9526-9531 (2014).
4. Wang, S. *et al.* Highly stretchable and self-healable supercapacitor with reduced graphene oxide based fiber springs. *ACS Nano* **11**, 2066-2074 (2017).
5. Guo, Y., Zheng, K. & Wan, P. A flexible stretchable hydrogel electrolyte for healable all-in-one configured supercapacitors. *Small* **14**, 1704497 (2018).
6. Guo, Y. *et al.* A self-healable and easily recyclable supramolecular hydrogel electrolyte for flexible supercapacitors. *J. Mater. Chem. A* **4**, 8769-8776 (2016).
7. Tao, F., Qin, L., Wang, Z. & Pan, Q. Self-healable and cold-resistant supercapacitor based on a multifunctional hydrogel electrolyte. *ACS Appl. Mater. Interfaces* **9**, 15541-15548 (2017).
8. Wang, Z., Tao, F. & Pan, Q. A self-healable polyvinyl alcohol-based hydrogel electrolyte for smart electrochemical capacitors. *J. Mater. Chem. A* **4**, 17732-17739 (2016).
9. Huang, Y. *et al.* A self-healable and highly stretchable supercapacitor based on a dual crosslinked polyelectrolyte. *Nat. Commun.* **6**, 10310 (2015).
10. Wang, Z. & Pan, Q. An omni-healable supercapacitor integrated in dynamically cross-linked polymer networks. *Adv. Funct. Mater.* **27**, 1700690 (2017).
11. Chen, C. R., Qin, H., Cong, H. P. & Yu, S. H. A highly stretchable and real-time healable supercapacitor, *Adv. Mater.* **31**, 201900573 (2019).
